# Team Dynamics in Hospital Workflows: An Exploratory Study of a Smartphone Task Manager

**DOI:** 10.2196/28245

**Published:** 2021-08-16

**Authors:** Danula Hettiachchi, Lachie Hayes, Jorge Goncalves, Vassilis Kostakos

**Affiliations:** 1 School of Computing and Information Systems The University of Melbourne Parkville Australia; 2 Nothern Hospital Epping Epping Australia

**Keywords:** task assignment, smartphones, hospital communication, clinical workflows, mobile app, clinical platform, mHealth

## Abstract

**Background:**

Although convenient and reliable modern messaging apps like WhatsApp enable efficient communication among hospital staff, hospitals are now pivoting toward purpose-built structured communication apps for various reasons, including security and privacy concerns. However, there is limited understanding of how we can examine and improve hospital workflows using the data collected through such apps as an alternative to costly and challenging research methods like ethnography and patient record analysis.

**Objective:**

We seek to identify whether the structure of the collected communication data provides insights into hospitals’ workflows. Our analysis also aims to identify ways in which task management platforms can be improved and designed to better support clinical workflows.

**Methods:**

We present an exploratory analysis of clinical task records collected over 22 months through a smartphone app that enables structured communication between staff to manage and execute clinical workflows. We collected over 300,000 task records between July 2018 and May 2020 completed by staff members including doctors, nurses, and pharmacists across all wards in an Australian hospital.

**Results:**

We show that important insights into how teams function in a clinical setting can be readily drawn from task assignment data. Our analysis indicates that predefined labels such as urgency and task type are important and impact how tasks are accepted and completed. Our results show that both task sent-to-accepted (*P*<.001) and sent-to-completed (*P*<.001) times are significantly higher for routine tasks when compared to urgent tasks. We also show how task acceptance varies across teams and roles and that internal tasks are more efficiently managed than external tasks, possibly due to increased trust among team members. For example, task sent-to-accepted time (minutes) is significantly higher (*P*<.001) for external assignments (mean 22.10, SD 91.45) when compared to internal assignments (mean 19.03, SD 82.66).

**Conclusions:**

Smartphone-based task assignment apps can provide unique insights into team dynamics in clinical settings. These insights can be used to further improve how well these systems support clinical work and staff.

## Introduction

The free availability, widespread use, reliability, and intuitive nature of modern communication interfaces have led to the use of popular messaging apps like WhatsApp among medical staff [1]. These apps can bring many benefits to clinical teams, such as prompt communication, reduction in interruptions, ability to form groups, and convenient access to other staff members [1,2]. Positive outcomes of using WhatsApp for clinical communication have been highlighted in numerous studies conducted among emergency surgery team members in a hospital in the United Kingdom [3], surgeons of two hospitals in Italy [4], orthopedic team members in a hospital in Ireland [5], and all professionals in medical and emergency teams in a major hospital in Malaysia [2].

However, using personal devices and generic messaging apps poses privacy and security concerns [6]. For example, when handling protected health information (PHI), medical professionals in the United States are required to adhere to communication regulations set out in the Health Insurance Portability and Accountability Act (HIPAA). Nevertheless, there is a lack of awareness and no consensus among medical staff on what apps are considered to be compliant with HIPAA [7]. Other adverse consequences of using regular messaging apps for work include information overload and the impact on the separation between one’s work and personal life [2,6].

Although such off-the-shelf messaging apps are ubiquitous and convenient [2], they do not provide structured task management features that would allow medical staff to send, accept, and prioritize tasks. Khanna et al [8] describe how a smartphone-based paging app can potentially bring numerous benefits to medical staff. Integrated interfaces can reduce the effort required to accept or send tasks. Notifications can streamline work by reducing the need to check for updates proactively. Such apps could also learn and initiate routine communications without human intervention to reduce redundant tasks. Similarly, Patel et al [9] report that mobile app–based communication improves efficiency, reduces interruptions, and allows health care professionals to transfer information reliably and clearly compared to using a standard pager system. Therefore, communication tools that overcome the aforementioned security challenges and provide smart task management capabilities are desirable within the health care industry.

In addition, a centralized tool for communication and task assignment can provide greater value for hospitals by providing the opportunity to analyze and understand the team dynamics of medical activity [10,11]. Literature presents such computer-based task management systems and their positive outcomes (eg, a desktop-based system implemented at Middlemore Hospital, Auckland, New Zealand [12], and a system that sends messages to dedicated team smartphones at Toronto General Hospital and Toronto Western Hospital, Toronto, Canada [13]). Similarly, Dock Health is a more modern team collaboration and task management app adopted by medical staff at Boston Children’s Hospital, Boston, MA, United States [14].

Although hospitals have begun to adopt smartphone-based task management and communication systems, it remains unclear how the task records can be used to gain a better understanding of medical team dynamics and communication patterns. Such insights can be critical in further improving the user experience of the app as well as increasing the efficiency of the task assignment process. Previous attempts to examine hospital workflows and staff communication include ethnographic methods and interviews with clinical staff [11,15], analyzing patient electronic medical records (EMRs) [16], and mapping call data [17]. However, it is costly and challenging to implement such methods at scale, and they fail to provide in-depth and timely data, unlike data collection through task management apps.

**Figure 1:**
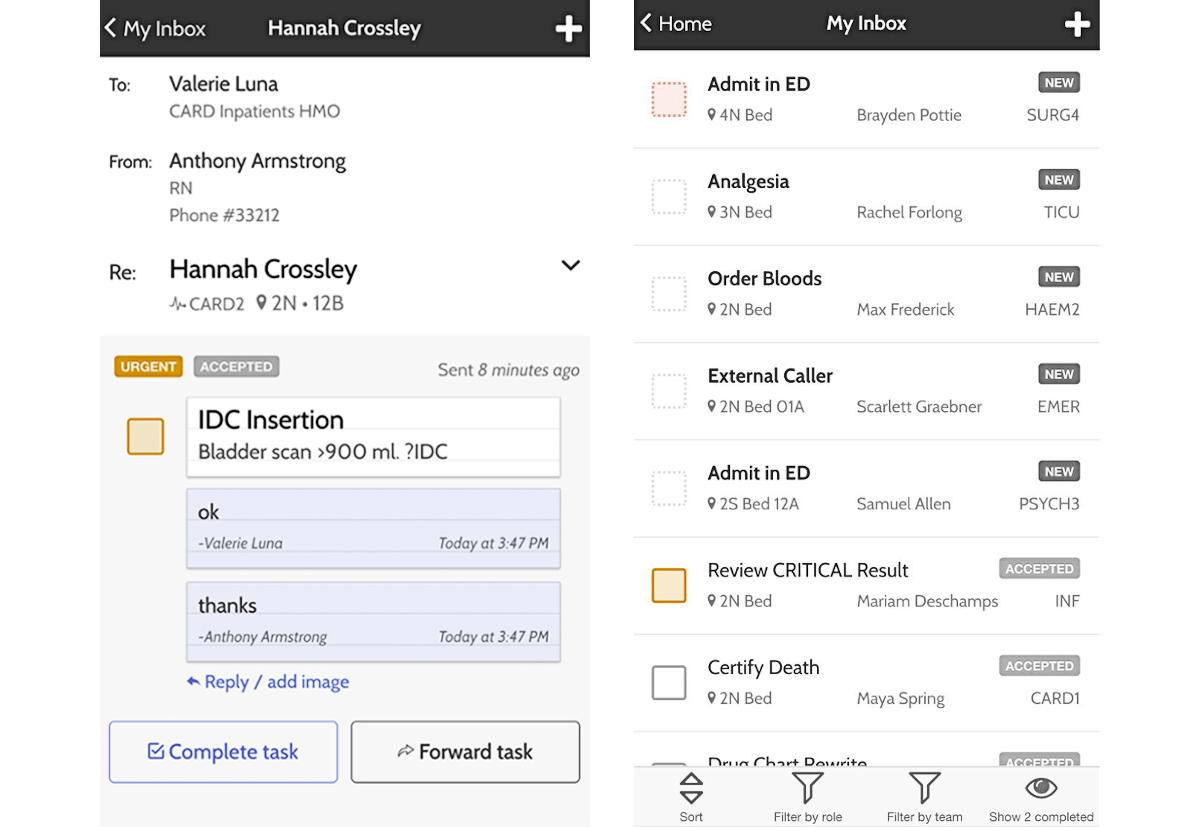
Examples of the MedTasker mobile app interface [18]. Names and task details are fictional.

In this paper, we present an exploratory analysis of medical task assignment data collected through a mobile app deployed at a hospital in Australia for 22 months. In our study, all staff at a hospital started using a bespoke smartphone platform, MedTasker [18] (Figure 1), to manage and execute clinical workflows. Tasks are defined as work units assigned to a specific staff member through our app and include tasks such as reviewing medications, admitting a patient to a ward, or conducting a medical procedure.

In our exploratory analysis, we seek to identify whether the increased granularity of the collected data provides insights into the hospital’s workflows (ie, repeatable patterns of clinical tasks). For example, we are interested in understanding the various individual and team dynamics (ie, factors that influence the direction of a team’s behavior and performance) that underpin workflows at the hospital. Furthermore, our analysis also seeks to identify ways in which task management platforms can be improved and designed to better support clinical workflows.

## Methods

### MedTasker App

MedTasker [18] is a mobile communication and task management platform built for hospitals. In addition to the typical task management features to create, send, accept, and forward tasks, MedTasker provides a staff directory and supports comments, notes, and attachments. The app includes an escalation process, which can alert additional staff members when the recipient does not accept tasks within a specified period. In addition, hospitals can configure the escalation process to tailor their needs.

The app can also integrate with existing hospital systems such as the Patient Administration System, EMRs, pathology, radiology, paging systems, and Active Directory. All communications using MedTasker use end-to-end encryption. The app also provides a way to share clinical images and files in a secure and privacy-compliant way. Since all tasks are tracked in real time, MedTasker enables visualizations to better manage team workloads and audit task workflows when needed.

### Data Collection

MedTasker has been used as the regular task management solution at Northern Hospital, Epping, Victoria, Australia, since 2018. Northern Hospital is the major public health care provider for acute, maternity, subacute, and specialist services in Melbourne’s northern suburbs and surrounding regional areas. The hospital has over 5300 dedicated professional staff and treats over 94,000 patients admitted yearly. All staff members including doctors, nurses, and pharmacists use the MedTasker app for general task management across all wards. The app is accessible through desktop and mobile devices (Android and iPhone). Staff typically use the app on their personal smartphones and connect to the internet through the hospital’s Wi-Fi network. We collected task assignment data through MedTasker for a period of 22 months starting from July 1, 2018. Data fields include recipient team and level, patient team, sender role, task type, history, urgency, and time. Individual sender and receiver details in the data set were anonymized.

### Preprocessing

We adopted several preprocessing steps to ensure the reliability of the data. First, we filtered a number of tasks that were completed immediately after accepting (ie, accepted-to-completed time is 0 minutes). These records mainly correspond to instances where the staff member has already completed the task by the time they mark it as accepted in the system. Second, we removed tasks where sent-to-accepted time exceeded 24 hours. We also excluded all the tasks that were not marked as completed (ie, incomplete tasks) by the end of the time window considered.

### Analysis

Our analysis used statistical packages in R (version 3.6.1, R Foundation for Statistical Computing). We used nonparametric tests, the Wilcoxon rank-sum test, and the Kruskal-Wallis rank-sum test when comparing the task acceptance time between different groups and conditions. Finally, our results were discussed in focus groups and interviews with key hospital members, who reflected on our findings and helped us interpret them. Focus groups and interviews took place at the start of the analysis, halfway through data analysis, and upon completion of the analysis. These sessions lasted about one hour and involved hospital members and the authors of the study.

We mainly used task acceptance time, task completion time, redirection percentage, and task escalation percentage for our analysis. The metric selection was informed by the input provided by the hospital staff; they regularly use these metrics to monitor and evaluate the effectiveness of the task assignment process.

## Results

### Task Creation

**Figure 2:**
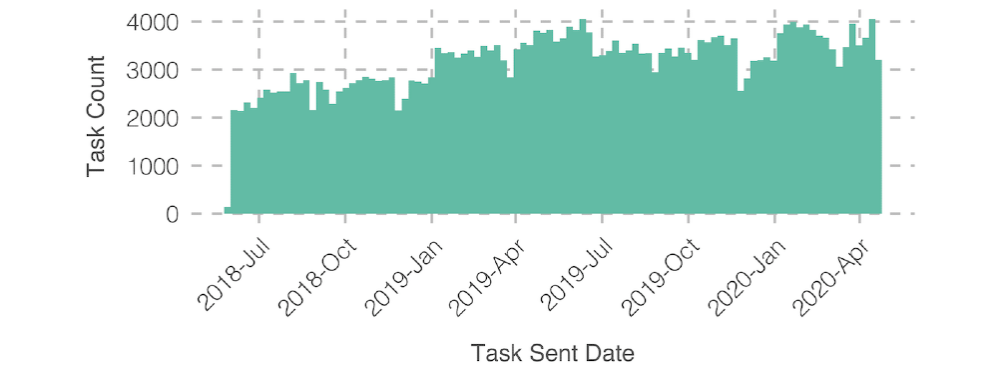
Tasks sent throughout the data collection period.

**Figure 3:**
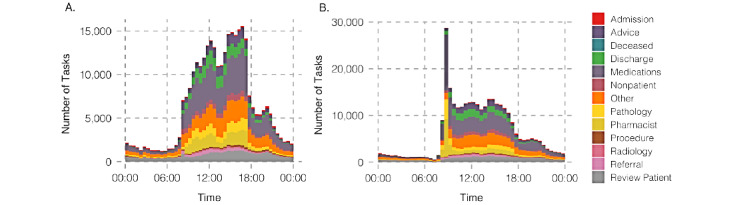
Task acceptance (A) and completion (B) throughout the day.

In total, 317,372 tasks were sent and completed between July 2018 and May 2020, with a mean sent-to-accepted time of 15.89 (SD 79.72) minutes. In Figure 2, we observe that the number of tasks sent through the MedTasker app gradually increased within the first year and maintained a consistent level thereafter. On average, 419.88 (SD 121.11) tasks were sent each day for the first 12 months. The average daily task count then increased to 501.83 (SD 123.25) for the remaining duration. Figure 3 shows how the time of day impacts task acceptance and behavior across different types of tasks.

### Task Urgency and Escalation

**Figure 4:**
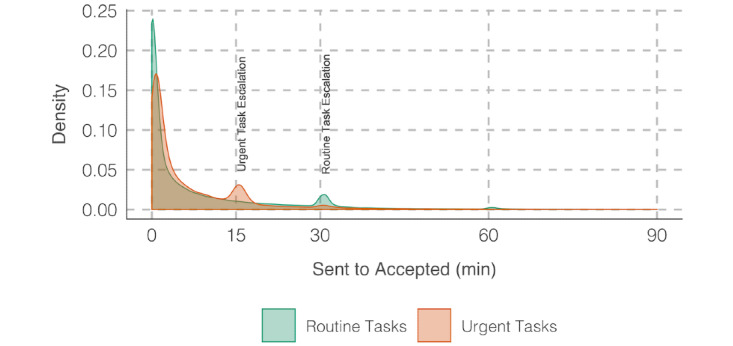
Variation in sent-to-accepted time across routine and urgent tasks.

A total of 13,168 of 317,372 (4.1%) tasks were categorized as urgent, and the remaining tasks were routine tasks. The mean sent-to-accepted time was 13.85 (SD 77.81) minutes for urgent tasks and 15.97 (SD 79.80) minutes for routine tasks. A Wilcoxon rank-sum test showed that sent-to-accepted time is significantly higher for routine tasks when compared to urgent tasks (*W*=1,911,381,996; *P*<.001), suggesting that recipients accept urgent tasks quicker than routine tasks. Figure 4 shows the impact of task urgency and escalation on sent-to-accepted time. Similarly, the sent-to-completed time (hours) was significantly higher (*W*=2,651,177,476; *P*<.001) for routine tasks (mean 14.74, SD 53.25) when compared to urgent tasks (mean 4.10, SD 17.84). The results also indicated that urgent tasks are more likely to be escalated when compared to routine tasks (*χ*^2^_1_=453.17; *P*<.001).

### Task Types

**Figure 5:**
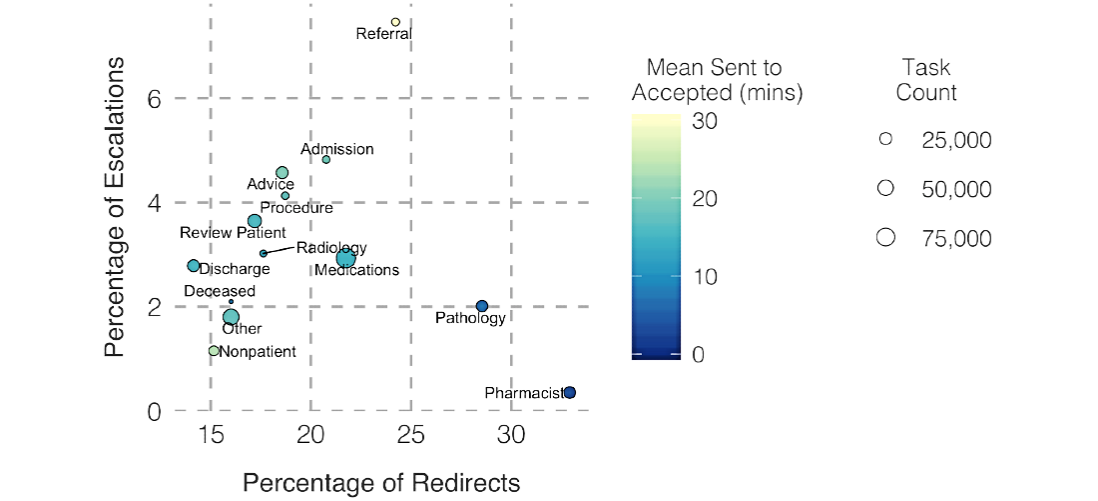
Task count, redirects, escalations, and mean sent-to-accepted time for different task types.

In Figure 5, we explore the variation in the percentage of redirects and escalated tasks against mean sent-to-accepted time using a high-level categorization of tasks. We note key deviations in task types such as Referral, Pathology, and Pharmacist.

### Patient and Recipient Team

**Figure 6:**
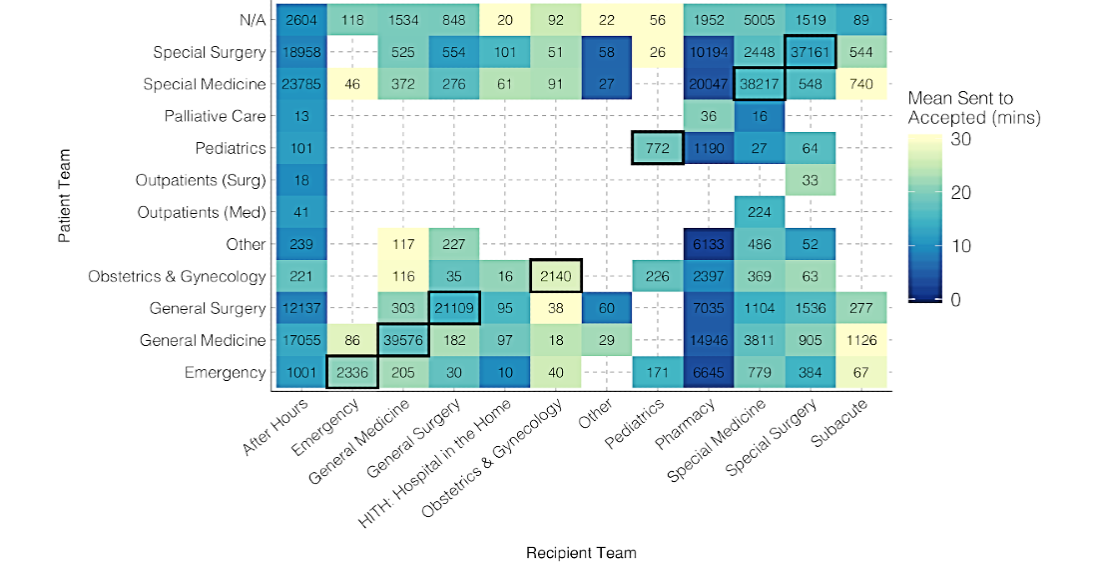
Task flow from patient teams to recipient teams. Med: medical; surg: surgical.

To analyze the impact of patient and recipient teams on task acceptance, we created a heat map visualization of task flow, where the total number of tasks are given in each cell (Figure 6). Cells with less than 10 tasks were removed from the graph. We observed that task acceptance times vary depending on the Patient (ie, Sender) and Recipient teams.

To further investigate the team dynamics in the hospital, we categorized tasks as “internal” versus “external” based on surgical and medical wards in the hospital. If the patient team and recipient team were the same for a particular task, then that task was labelled as an internal task. Tasks originating from or received by teams that do not belong to either the medical or surgical categories were excluded from this analysis. A Wilcoxon rank-sum test showed that the sent-to-accepted time (minutes) is significantly higher (*W*=441,094,646; *P*<.001) for external assignments (mean 22.10, SD 91.45) when compared to internal assignments (mean 19.03, SD 82.66). Similarly, the sent-to-completed time (hours) was significantly higher (*W*=457,480,292; *P*<.001) for external assignments (mean 12.72, SD 42.71) when compared to internal assignments (mean 6.17, SD 22.90). We also observed a task escalation rate of 5.01% in external tasks and 3.45% in internal tasks. A chi-square test showed that external tasks are more likely to be escalated when compared to internal tasks (*χ*^2^_1_=38.72; *P*<.001).

### Impact of Roles

The majority of tasks were received by hospital medical officers (HMOs; 119,582/317,372; 37.7%) and interns (94,869/317,372; 29.9%), whereas registrars received a smaller portion of tasks (23,041/317,372; 7.3%). In terms of senders, the majority of tasks were sent by nurses (187,487/317,372; 59.1%). Table 1 shows the variation in task acceptance across different recipient groups.

**Table 1:** Impact of the recipient level.

Recipient level	Mean sent-to-accepted time (minutes)	Mean sent-to-completed time (hours)	Redirects, %	Escalations, %
Intern	17.91	5.22	15.49	2.98
Hospital medical officer	15.60	4.54	15.83	2.87
Registrar	29.36	16.97	32.63	6.58

## Discussion

### MedTasker and Task Assignment

Task assignment and management is an important workflow aspect in hospitals that is not well supported by popular communication tools. In our work, we studied the use of MedTasker, a purpose-built task assignment app, and analyzed task assignment data collected over a period of over 22 months. As suggested through the usage trends in Figure 2, hospital staff comfortably adopted the MedTasker app within a year. Task acceptance and completion patterns throughout the day are proportionate to staff availability and reflect the standard hospital schedule where nurses change shifts at 7 AM, 3 PM, and 11 PM. Interestingly, we noted a high number of tasks being accepted at the beginning of the day shift (Figure 3). Although the total number of tasks accepted generally declines through the day, the number of tasks completed increases until the day shift ends.

Our analysis considers the tasks’ sent-to-accepted time and the percentage of tasks that are escalated or redirected as key metrics in our evaluation. The analysis indicates significant variations in these important task metrics when we consider task type, task urgency, team, and sender roles. These variations highlight how task assignment data collected through MedTasker reflects the existing operational realities of a clinical environment.

### Team Dynamics and Communication in the Hospital

A central theme that emerged from our results and interviews is the trust within and across teams. When examining task types and corresponding metrics in Figure 5, we observe different working patterns. Pharmacist and Pathology tasks have higher task redirection rates, indicating that task senders are not assigning them to the right person in the first instance. However, lower mean sent-to-accepted times suggest that such tasks are quickly redirected to a relevant team member and then accepted. This demonstrates how the specific teams that undertake these tasks function as efficient teams. In contrast, other core medical tasks like Review patient and Procedure are directed to the right person but are not accepted as fast as tasks like Pathology and Pharmacist. Core medical tasks also have relatively high escalation levels. Naturally, it is difficult to sort many core medical tasks into a well-defined task category as they could overlap with multiple categories. These different task acceptance paradigms also highlight the separation between tasks intended for a specific person (eg, Review patient) and tasks intended for a specific team (eg, Pathology). We observe the need for the software to support well-defined tasks and roles, as well as for the hospital to have closely functioning teams to achieve high efficiency in clinical settings. Referral tasks appear as an outlier with high mean sent-to-accepted time, escalations, and redirections compared to other tasks. Although they are tasks sent among medical staff, they are generally treated as nonurgent tasks.

We obtained further insights about team dynamics by considering the task flow between different patient and recipient teams. Generally, we observed reasonable mean sent-to-accepted times across the majority of the team interactions while certain teams exhibit specific patterns. For example, we observed relatively higher mean sent-to-accepted times for tasks sent and received by the Obstetrics and Gynecology team (Figure 5). This team was slightly underresourced and stationed at a separate ward that is not well-connected with the rest of the hospital, decreasing the trust between teams. In addition, the Pharmacy team stands out with better mean sent-to-accepted times due to their tasks not being generally directed at a specific person. Another key observation is the separation between surgical and medical teams. Our results show that internal tasks or tasks sent among medical or surgical teams have significantly faster task acceptance and completion times and fewer escalations when compared to external tasks or tasks sent across teams. These observations highlight the need to facilitate intrateam and interteam connections and trust for operational efficiency.

Similarly, in terms of sender and recipient roles, we found higher redirect and escalation percentages and longer task acceptance times for tasks received by registrars in comparison to junior staff such as interns and HMOs (Table 1). This observation is expected since Registrars are experienced staff members, and tasks accepted by them are more complex and require expertise. In addition, the findings suggest that HMOs, who received the largest portion of tasks (119,582/317,372; 37.7%), are better organized and more efficient compared to others.

### Redesigning Task Management Apps

Computer-based task management systems have the potential to benefit junior medical officers and nurses by improving overall task communication and achieving large reductions in time spent dealing with requests and walking between wards [19]. Early work on desktop computer–based task management systems includes TaskManager [12], a system implemented at Middlemore Hospital, Auckland, New Zealand. Their study shows that having the task management application connected to the hospital’s Patient Management System increases the ease of task creation and results in effective communication. Similarly, a communication system deployed at Toronto General Hospital and Toronto Western Hospital, Toronto, Canada, involves a desktop-based physician handover tool and an SMS-based system that sends secure messages to a dedicated team smartphone [13]. A subsequent survey found that clinicians perceive that the system has a positive impact on efficiency and helped speed up daily work tasks. A more modern solution is Dock Health [14], a team collaboration and task management app that has been successfully adopted by medical staff at Boston Children’s Hospital, Boston, MA, United States. Their HIPAA-compliant app runs on both mobile devices and web browsers and aims to overcome typical design and user experience issues in health care software.

In our case, our analysis has focused on a particular platform—MedTasker—but nevertheless, our analysis shows more broadly the kinds of meaningful insights that can be derived from data logs of task management apps. These insights can drive policy changes, which in turn can increase productivity in hospitals. Large volumes of historic data can also be used to build smart task management solutions that can optimally schedule tasks and alert when there are resource shortcomings. Additionally, important employee well-being surveys or feedback elements can be easily integrated into the task assignment app. Unlike when using off-the-shelf communication apps such as WhatsApp, by using an app like MedTasker, the hospital administration can have control over how data is governed and avoid security and privacy irregularities [6]. In addition, smartphones apps can be used to effectively communicate with and educate patients [20]. Such apps can be seamlessly integrated with task management apps. These unique advantages make purpose-built task assignment apps like MedTasker very appealing. We also point out that other forms of communication can complement app usage. In our case, hospital staff mentioned using phone calls for extended detailed conversations that mainly involve administrative work, WhatsApp for social collaboration and to notify regarding nonclinical events, and paging systems or speaker announcements for emergencies. Face-to-face communication also regularly occurs within wards but was not captured in this study.

Based on our observations and discussions with hospital staff, we discuss several improvements to MedTasker that we aim to implement in the future. These enhancements are also important to consider when implementing similar task assignment apps for health care. First, our results show that reminders have a strong influence on task acceptance. As opposed to using static time limits, adaptive time limits can be used to send reminders. It is also possible to incorporate workload information, such that reminders are adjusted based on recipients’ ongoing workload.

Second, the current practice of creating a task with an urgent or nonurgent label is arbitrary and highly dependent on the individual who creates the task. Since the prespecified task urgency has a significant impact on task acceptance, it is important that this particular label is added appropriately. Future implementations could help task requesters by automatically suggesting the appropriate urgency label based on task information. In addition, we propose user interface improvements that direct attention to urgent tasks when users receive multiple tasks.

Third, our results suggest that trust is important for efficient task assignment. To facilitate trust among teams and individuals, task assignment apps could provide more information regarding users. Contextual information such as current workload information or location can be helpful. Profile photos and other elements are also useful in increasing the levels of image appeal and perceived social presence, which in turn can increase trust [21].

Furthermore, MedTasker is not currently integrated with My Health Record (a major national electronic health record initiative in Australia) or any other external systems such as insurance systems. Although such integrations can be facilitated to further improve patient care, they should be implemented based on policy decisions and guidelines provided by hospitals and regulators.

### Limitations

We note several limitations in our work. First, we use task acceptance time and completion time from the data set in our analysis. However, there may be some tasks in which acceptance and completion times recorded through the app do not correspond to actual task times. For example, staff may not immediately indicate task completion when they attend to a series of tasks or experience internet connectivity issues. Second, we acknowledge that different hospitals may operate under different protocols and practices, and as such, it is important to be cautious about extrapolating our findings to other or all hospitals, especially in different countries.

### Conclusion

We analyzed hospital task assignment data collected via MedTasker, a dedicated task assignment app deployed at a hospital over 22 months. We show that important insights into how teams function in a clinical setting can be readily drawn from task assignment data. Our analysis shows that predefined labels such as urgency and task type are important and impact how tasks are accepted and completed. We also show how task acceptance varies across teams and roles and highlight that internal tasks are more efficiently managed than external tasks, possibly due to increased trust among team members. Finally, we discuss how smartphone-based task assignment apps can be further improved to support clinical work and staff.

## Abbreviations

EMR: electronic medical record
HIPAA: Health Insurance Portability and Accountability Act
HMO: hospital medical officer
PHI: protected health information
